# Genome-Wide Association Study Identifies *Phospholipase C zeta 1 (PLCz1)* as a Stallion Fertility Locus in Hanoverian Warmblood Horses

**DOI:** 10.1371/journal.pone.0109675

**Published:** 2014-10-29

**Authors:** Rahel Schrimpf, Claudia Dierks, Gunilla Martinsson, Harald Sieme, Ottmar Distl

**Affiliations:** 1 Institute for Animal Breeding and Genetics, University of Veterinary Medicine Hannover, Hannover, Germany; 2 State Stud Celle of Lower Saxony, Celle, Germany; 3 Clinic for Horses, Unit for Reproduction Medicine, University of Veterinary Medicine Hannover, Hannover, Germany; University of Sydney, Australia

## Abstract

A consistently high level of stallion fertility plays an economically important role in modern horse breeding. We performed a genome-wide association study for estimated breeding values of the paternal component of the pregnancy rate per estrus cycle (EBV-PAT) in Hanoverian stallions. A total of 228 Hanoverian stallions were genotyped using the Equine SNP50 Beadchip. The most significant association was found on horse chromosome 6 for a single nucleotide polymorphism (SNP) within *phospholipase C zeta 1 (PLCz1)*. In the close neighbourhood to *PLCz1* is located *CAPZA3* (*capping protein (actin filament) muscle Z-line*, *alpha 3*). The gene *PLCz1* encodes a protein essential for spermatogenesis and oocyte activation through sperm induced Ca^2+^-oscillation during fertilization. We derived equine gene models for *PLCz1* and *CAPZA3* based on cDNA and genomic DNA sequences. The equine *PLCz1* had four different transcripts of which two contained a premature termination codon. Sequencing all exons and their flanking sequences using genomic DNA samples from 19 Hanoverian stallions revealed 47 polymorphisms within *PLCz1* and one SNP within *CAPZA3*. Validation of these 48 polymorphisms in 237 Hanoverian stallions identified three intronic SNPs within *PLCz1* as significantly associated with EBV-PAT. Bioinformatic analysis suggested regulatory effects for these SNPs via transcription factor binding sites or microRNAs. In conclusion, non-coding polymorphisms within *PLCz1* were identified as conferring stallion fertility and *PLCz1* as candidate locus for male fertility in Hanoverian warmblood. *CAPZA3* could be eliminated as candidate gene for fertility in Hanoverian stallions.

## Introduction

Stallion fertility is of increasing importance due to the widespread use of artificial insemination in horse breeding and the strong seasonal breeding restricted to six months of the year. Studies in human and mice discovered a large number of genes with influence on male fertility [1]–[5], whereas there are only few reports about genes with impact on stallion fertility [6]–[10]. To date, genome-wide association studies (GWAS) with dense genotyping arrays greatly enhance the possibilities to clarify the role of known candidates and to identify new promising candidate genes for mammalian fertility [11]–[13].

Several studies implicated candidate genes for stallion fertility that have been shown to play a substantial role in equine male reproduction. In Hanoverian stallions, single nucleotide polymorphisms (SNPs) within the genes *prolactin receptor* (*PRLR*) [6], *inhibin beta A (INHBA)* [7], *follicle stimulating hormone* (*FSHB*) [8], *angiotensin converting enzyme* (*ACE*) [8], *spermatogenesis associated 1 (SPATA1)* [9] and *cysteine-rich secretory protein 3 (CRISP3)* [10] were shown significantly associated with stallion fertility. Recently, a GWAS employed in seven Thoroughbred stallions with impaired acrosome reaction (IAR) and 37 controls suggested equine *FK506 binding protein (FKBP6)* as an IAR-susceptibility locus in Thoroughbred stallions [14].

The objectives of the present study were to perform a GWAS for stallion fertility in Hanoverian warmblood horses. Estimated breeding values of the paternal component of the pregnancy rate per estrus cycle (EBV-PAT) were employed as the target trait for stallion fertility in Hanoverian warmblood horses. The most significant association for stallion fertility was found for a single nucleotide polymorphism (SNP) within the gene *phospholipase c zeta (PLCz1)* on horse chromosome (ECA) 6. Close to *PLCz1*, *capping protein (actin filament) muscle Z-line*, *alpha 3* (*CAPZA3*) was identified as a second potential candidate gene. We sequenced *PLCz1* and *CAPZA3* using cDNA and genomic DNA samples to construct the gene models and to perform mutation detection. Validation of the 48 polymorphisms identified within *PLCz1* and *CAPZA3* was performed in 237 Hanoverian stallions.

## Results

### Genome-wide association

**Figure 1:**
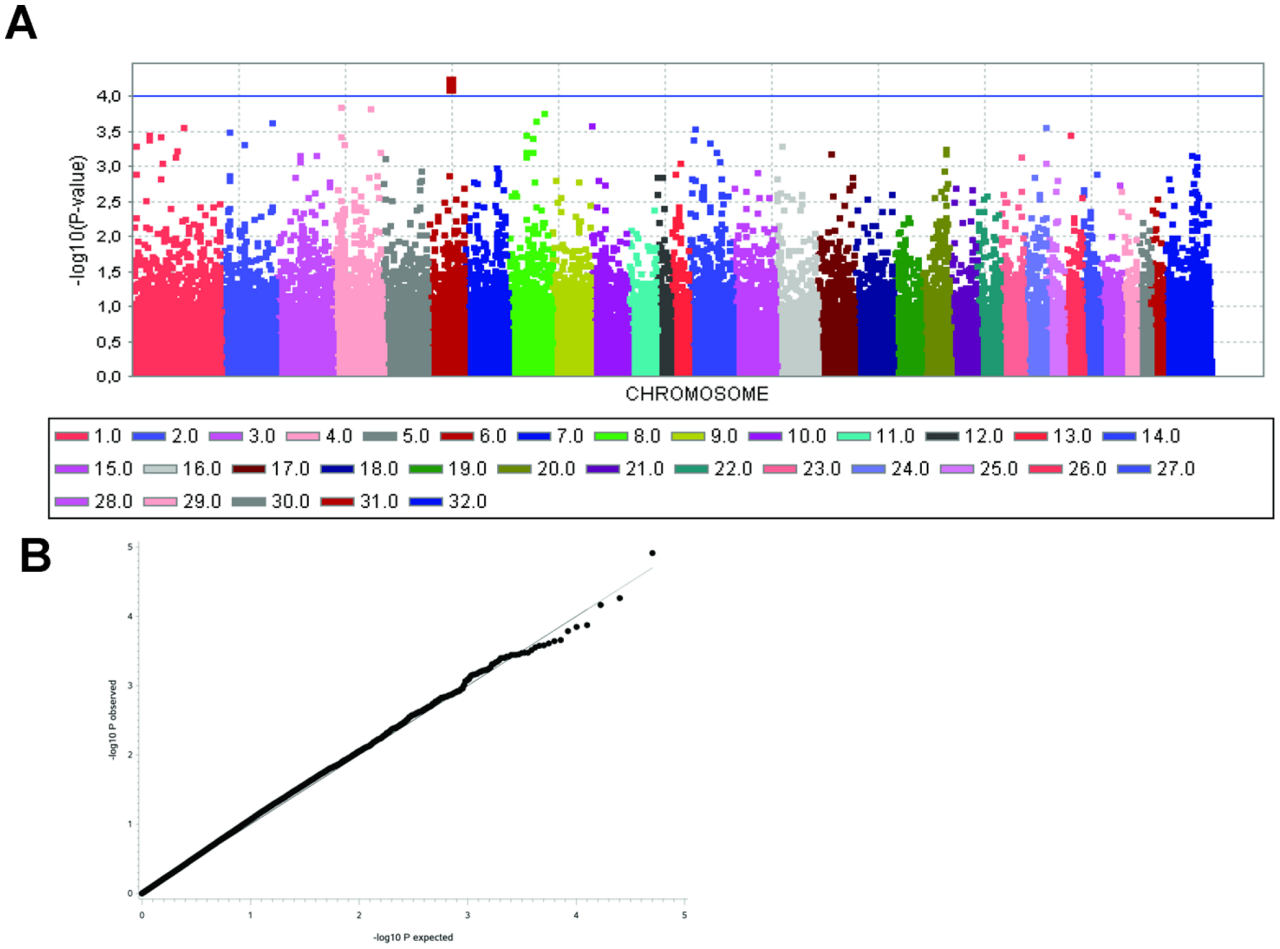
Manhattan plot of −log_10_P-values of the genome-wide association study for estimated breeding values of the paternal component of the pregnancy rate per estrus cycle (EBV-PAT) in Hanoverian stallions using a mixed linear model analysis (A) and Q-Q plots of observed versus expected −log_10_P-values (B) from the mixed linear model analysis for EBV-PAT. (A) On the X-axis, the SNPs are given by horse chromosome number. The −log_10_P-values for each SNP effect are plotted against the SNP position on each chromosome. Chromosomes are differentiated by colors. The color keys are given below the plot. The blue line indicates the threshold of the −log_10_P-values >4.0. The peak value (BIEC2-952439) is located on horse chromosome 6 at 45.556 Mb. (B) The expected −log_10_P-values (solid line) are plotted against the observed −log_10_P-values (black dots).

In 228 Hanoverian warmblood stallions, the genome-wide association study (GWAS) for stallion fertility using a mixed linear model (MLM) analysis revealed the highest association for the genomic region at 45,571,963–45,612,102 base pairs (bp) on ECA6 for EBV-PAT (Figure 1A). Within this region, the highest associated SNP BIEC2-952439 (g.45586821C>T) reached a −log_10_P-value of 4.14 and explained 6.72% of the variance of EBV-PAT. Within this region, seven protein-coding genes, eight pseudogenes und two non-coding RNAs are annotated using the horse genome reference assembly EquCab2.0 (http://www.ensembl.org/Equus-caballus/) (Figure S1). We located the SNP BIEC2-952439 within intron 8 of the *phospholipase c zeta 1 (PLCz1)* gene, annotated at 45.571–45.612 Mb. Downstream to *PLCz1*, the gene *capping protein (actin filament) muscle Z-line*, *alpha 3* (*CAPZA3*) is annotated at 45.612–45.614 Mb. The observed −log_10_P-values were plotted against the expected −log_10_P-values and the quantile-quantile (Q-Q) plots illustrated that the population stratification was eliminated through the model employed (Figure 1B). Analysis of the haplotype structure of the SNP BIEC2-952439 flanking genomic region identified 15 haplotype blocks. Haplotype block 9 at 45,402,588–45,586,821 bp extends over 194 kb and includes the genome-wide significantly associated SNP BIEC2-952439 (Figure S1).

### Sequencing of *PLCz1* and *CAPZA3*

**Figure 2:**
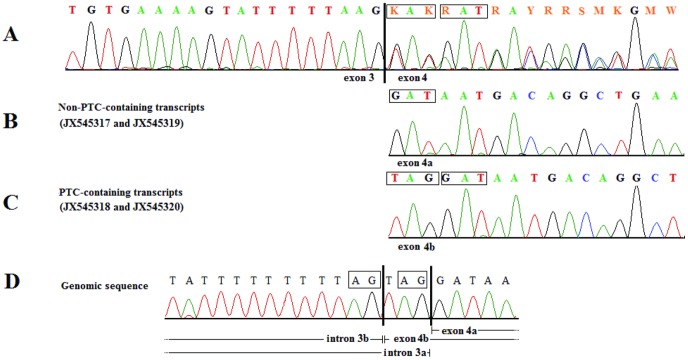
Chromatograms of transcripts detected in equine *PLCz1*. (A) The sequences of all animals tested show both adjacent splice sites of the cDNA. Both, forward and reverse sequences show ambiguous double traces when elongation passes exon boundary. (B, C) Visualization of single trace sequence is used to portray each single transcript. (B) For the transcripts without a premature termination codon (PTC), exon 4a starts with the residues GAT, (C) whereas PTC-containing transcript variants harbor an extended exon 4b and start with the residues TAG. (D) Genomic sequence was used to illustrate usage of adjacent acceptor splice sites AG-TA for PTC-containing transcripts and AG-GA for non-PTC-containing transcripts. Vertical lines mark the exon/exon boundary or intron/exon boundary, respectively. The black open boxes indicate specific nucleotide residues used.

**Figure 3:**
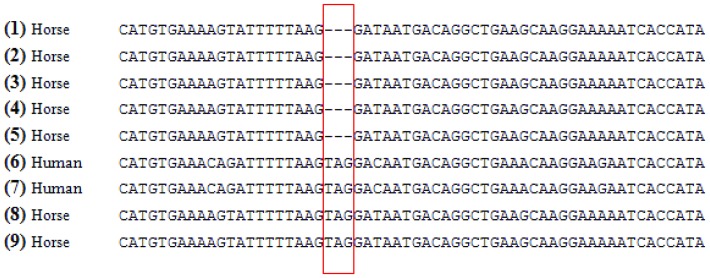
Comparison of equine *PLCz1* transcript variants with and human *PLCz1*. Transcript variants (1) ENSECAT00000012304, (2) ENSECAT00000012372, (3) XM_001497766.3, (4) JX545317, (5) JX545319, (6) ENST00000318197, (7) NR_073075.1, (8) JX545318, and (9) JX545320 are presented. (1–4) Human and equine variants without a premature termination codon (PTC) in sequences are indicated. (5–9) A PTC is activated within human and equine variants at similar positions (c.136). The PTC region is highlighted by a red box.

We selected *PLCz1* and *CAPZA3*, alias LOC100067825, for re-sequencing because intron 8 of *PLCz1* contained the EBV-PAT-associated SNP BIEC2-952439 and *CAPZA3* was the second candidate gene in this region, approximately 27 kb downstream to BIEC2-952439. We detected four transcript variants of the equine *PLCz1* in cDNA amplicons from testis tissue of each of the six stallions (Table 1, Figure S2). Each two transcripts differed in the size of the first untranslated exon. Transcripts JX545317 and JX545318) cover a smaller exon 1 (exon 1a) with 114 bp, whereas transcripts JX545319 and JX545320 show an extended exon 1 (exon 1b) containing 174 bp (Figure S3). Two transcripts (JX545318 and JX545320) contained a premature termination codon (PTC). The PTC resulted from the use of alternative acceptor splice sites at the boundary between intron 3 and exon 4 (Figure 2). Bioinformatic analyses using NetGene2 [15] and HSF 2.4.1 [16] for splice site prediction supported the existence of two adjacent acceptor splice sites at this intron-exon-boundary. For the non-PTC-containing transcripts (JX545317 and JX545319), the acceptor site was predicted with a reliability of 86% (HSF 2.4.1) and 97% (NetGene2). The acceptor site for the PTC-containing transcripts reached a reliability of 82% (HSF 2.4.1) and 63% (NetGene2). The PTC position at the exon-exon-junction (136 bp downstream to the translation start) identified in the equine *PLCz1* is identical to the PTC position in human (Figure 3).

**Table 1:** Transcript variants detected in the equine *PLCz1*.

Transcript variant ID	Exon 1	PTC	mRNA	CDS	Amino acids
JX545317	114	-	2220	1917	638
JX545319	174	-	2259	1917	638
JX545320	174	5′ exon 4	2262	1605	534
JX545318	114	5′ exon 4	2223	1605	534
ENSECAT00000012304	not annotated	-	2088	1917	638
XM_001497766	not annotated	-	2092	2049	682

Variants were detected in cDNA sequences of testis tissues from six Hanoverian stallions. The size of the untranslated exon 1, mRNA and coding sequence (CDS) in base pairs, the location of the premature termination codon (PTC) and the predicted number of amino acids are given.

The non-PTC-containing transcripts (JX545317 and JX545319) showed an open reading frame (ORF) of 1917 bp. For PTC-containing transcripts, we performed an *in-silico* analysis using ATG_PR_ for prediction of ORFs. Based on this analysis, two possible ORFs were distinguished. The first ORF (ORF1) predicted with a very low probability (0.06%) encodes a 45 amino acid (aa) containing protein. In human, at a similar position a truncated protein of 45 aa is predicted to elicit nonsense-mediated mRNA decay (NMD) (Figure S4). Due to the very low probability for the predicted ORF1 according to the *in-silico* analysis, we assumed that a protein resulting from ORF1 deems to be non-existent in horses. The second ORF (ORF2) was predicted with a probability of 25% to contain 1605 bp and to encode a protein of 534 aa. The usage of the alternative splice site extended exon 4 (exon 4b) by three base pairs (Figure S1). Translation start for ORF2 is located at the 3′end of exon 4 and translation termination is consistent with the non-PTC-containing transcripts.

### Mutation detection

Several mutations within *PLCz1* were reported to affect human male fertility [17]. *PLCz1* is involved in male fertility by triggering [Ca^2+^] oscillation during fertilization [18]. *CAPZA3* localized predominantly in the neck region of ejaculated sperm may play a role in sperm architecture and male fertility in man [19] and mice [20], [21]. Polymorphism detection was performed using genomic DNA of 19 fertile Hanoverian stallions with low (n = 6), moderate (n = 6), high (n = 3) and very high (n = 4) EBV-PAT (Table S1). Re-sequencing of all exons and exon-intron boundaries of *PLCz1* and *CAPZA3* revealed a total of 46 SNPs, whereof 45 were located within *PLCz1* and one within *CPAZA3* (Table S2). In addition, two intronic indels were detected (g.45610678delA and g.45581388delTTAA) within *PLCz1*. Validation of exonic mutations was done using the cDNA sequences of the six Hanoverian stallions employed for the analysis of the gene models. All exonic mutations could be identified in these six animals. In addition, to preclude effects of the indels on cDNA sequence, we verified the two intronic indels in three out of six of these animals.

**Figure 4:**
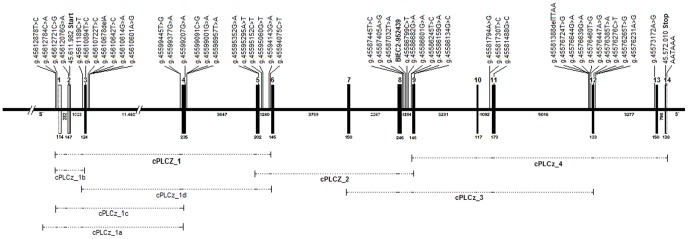
Equine gene model of *PLCz1* and polymorphisms detected. Sequence analyses of *PLCz* 1 revealed four transcripts of which the full-length variant (JX545317) is shown. Translated exons are shown as solid black boxes, untranslated exons as open boxes. Numbers above indicate the exon number. Continuous lines represent introns, numbers below the exons and introns indicate the sizes in base pairs. The predicted translation start and stop and the polyadenylation site is indicated. The highly associated single nucleotide polymorphism (SNP) BIEC2-952439 identified in a genome-wide association study (GWAS) is given in bold. The dashed lines below represent the coverage of the complementary DNA (cDNA) primers.

Within *PLCz1*, we identified 40 novel SNPs and confirmed five SNPs previously annotated by the Broad Institute (http://www.broadinstitute.org/ftp/distribution/horse_snp_release/v2/). Out of these 45 SNPs, eight were exonic, 33 intronic, one in the 5′UTR and three in the 5′ promoter region (Figure 4). In total, four exonic SNPs were missense mutations. A missense mutation within exon 9 (g.45586134G>C) caused an exchange of glutamine with glutamic acid (p.Gln400Glu) and had been classified as possibly damaging using PolyPhen-2 [22]. All other missense mutations were classified as benign. Within *CAPZA3*, a missense mutation (p.Leu259Pro) was detected. This mutation had been classified as probably damaging using PolyPhen-2 [22].

### Association analysis of candidate gene-associated polymorphisms

Association analysis in the detection sample (n = 19) revealed six SNPS and one indel significantly associated with EBV-PAT within *PLCz1* (Table S3). No EBV-PAT-associated SNP was found within *CAPZA3*. The minor allele frequencies (MAF) of the polymorphisms were at 0.05–0.37. Nominal P-values were at 0.015–0.05.

### Validation of candidate gene-associated polymorphisms

In total, we genotyped 16 polymorphisms and imputed the remaining 32 SNPs in 237 Hanoverian stallions. In order to genotype all polymorphisms possibly associated with EBV-PAT, we performed three rounds of genotyping and imputation. First, we genotyped the seven polymorphisms significantly associated in the detection sample and in addition, two synonymous and two non-synonymous exonic SNPs including the *CAPZA3*-related SNP. Then, we imputed the genotypes of the remaining 37 SNPs and performed an association analysis for all 48 polymorphisms. In the second round, we genotyped 13 polymorphisms and imputed 35 SNPs and in the third step, genotypes of 16 SNPs were available and imputed the genotypes of 31 SNPs. After each round, the SNPs with imputed genotypes and highest associations with EBV-PAT were genotyped. In the final association analysis, 14 polymorphisms were significantly associated with EBV-PAT (Table 2). After accounting for multiple testing using a Bonferroni correction, two genotyped SNPs (g.45599001G>A, P_Bonf_ = 0.047 and BIEC2-952439 (g.45586821C>T), P_Bonf_ = 0.047) and one imputed SNP (g.45595060G>T, P_Bonf_ = 0.037) reached significant associations with EBV-PAT.

**Table 2:** Significant association for 14 *PLCz1*-polymorphisms with estimated breeding values of the paternal component of the pregnancy rate per estrus cycle (EBV-PAT) in 237 Hanoverian stallions.

Polymorphism	MAF	PIC	HET	P-HWE	R^2^	P-Allele	P-Genotype	P-Bonf
g.45595060G>T	0.148	0.368	0.397	0.005	5.31	0.001	0.003	0.047
g.45599001G>A	0.238	0.297	0.401	0.110	6.40	0.002	0.001	0.047
g.45581730T>C	0.046	0.085	0.093	0.454	3.95	0.004	0.004	
g.45586682G>A	0.439	0.371	0.481	0.719	3.03	0.011	0.040	
g.45576724T>G	0.435	0.371	0.473	0.554	2.83	0.013	0.049	
g.45599377G>A	0.205	0.273	0.367	0.049	5.03	0.018	0.005	
g.45594075C>T	0.217	0.282	0.367	0.223	5.03	0.021	0.005	
g.45586821C>T	0.247	0.303	0.376	0.878	6.45	0.027	0.001	0.047
g.45581794A>G	0.232	0.293	0.380	0.314	3.84	0.031	0.016	
g.45581388delTTAA	0.232	0.293	0.329	0.238	2.47	0.032	0.072	
g.45586245T>C	0.458	0.373	0.511	0.662	3.99	0.033	0.014	
g.45576460T>G	0.006	0.013	0.013	0.922	1.88	0.047	0.046	
g.45586601G>A	0.460	0.373	0.515	0.577	4.80	0.069	0.006	
g.45612878T>C	0.030	0.056	0.042	0.0001	4.60	0.067	0.008	

For each polymorphism, minor allele frequency (MAF), polymorphism information content (PIC), heterozygosity (HET), test for Hardy Weinberg equilibrium (HWE) with the respective P-value (P-HWE), variance explained by the polymorphism for EBV-PAT (R^2^) and P-values for allelic (P-Allele) and genotypic (P-Genotype) association as well as after Bonferroni correction for multiple testing (P-Bonf) are given.

### Haplotype association for SNPs within *PLCz1*

In the subsequent haplotype analysis, we identified seven haplotype blocks using Haploview 4.2 [23] and the four gamete rule algorithm. Pairwise linkage disequilibria (r^2^) varied widely, but 24 SNPs reached r^2^-values >0.80 (Figure S5). Four adjacent haplotype blocks (block 2-block 5) were significantly (P = 0.005) associated with EBV-PAT (Table S4). The three significantly EBV-PAT-associated SNPs were contained in these haplotype blocks. Together, these haplotype blocks explained 17.36% of the variance (R^2^) of EBV-PAT in Hanoverian stallions. Variances explained by single haplotype blocks were at 4–11% (Table 3).

**Table 3:** Haplotype blocks within *PLCz1* significantly associated with estimated breeding values for the paternal component of the pregnancy rate per estrus cycle (EBV-PAT) in Hanoverian stallions.

Haplotype block	POS	SNP	R^2^ (%)	P
Block 2- Block 5	45,586,245–45,599,091		17.36	0.005
Block 2	45,586,245–45,586,601		4.09	0.013
Block 3	45,586,682–45,594,075	BIEC2-952439	10.88	0.002
Block 4	45,594,143–45,595,060	g.45595060G>T	4.86	0.015
Block 5	45,595,352–45,599,091	g.45599001G>A	7.69	0.011

The start and end of the haplotype block in base pairs (POS), the variance explained (R^2^) by each haplotype block, significantly associated SNPs within haplotype blocks (SNP) and P-values (P) are given.

## Discussion

Herein, we report a successful application of Equine SNP50 Genotyping Beadchip and GWAS for stallion fertility, implicating equine *PLCz1* as a locus for stallion fertility. The haplotype analysis corroborated the associations for single SNPs. We assume that several SNPs within *PLCz1* may have effects on trait expression and particularly, SNPs within the significantly associated haplotype blocks may be important for EBV-PAT in Hanoverian stallions. In comparison to the previous candidate gene studies [6]–[10], the variance explained by single *PLCz1*-associated SNPs and haplotypes was much larger. This may indicate the higher power of genome-wide studies contrasted to candidate gene studies. The associated SNPs do not influence the coding sequence of *PLCz1*, but may exhibit regulatory functions. Intronic mutations can affect transcription factor binding sites (TF) [24] and may cause significant phenotype diversity [25]. The highest associated SNP BIEC2-952439 influences the TF for estradiol receptor-alpha and -beta (ER-alpha, ER-beta). ER-alpha and ER-beta activated by estradiol influence male sperm metabolism and are involved in the pathophysiology of varicocele-associated male infertility [26]. Furthermore, estradiol exerts its cellular effects through estrogen receptors and acts as a germ cell survival factor in the human testis [27]. We propose, therefore, that intronic *PLCz1* SNPs associated with EBV-PAT may have regulatory functions via TFs and transcription levels of *PLCz1*.

Testis-specific mammalian *PLCz1* occupies an important key role in the cascade of male fertility. During fertilization sperm-induced [Ca^2+^] oscillation is essential for oocyte activation and support of embryonic development [28]–[30]. In mammals, this highly conserved mechanism [31]–[35] is triggered by PLCz1 released from the sperm during fertilization [28], [36]. In infertile men, defects in expression and abnormal patterns of PLCz1 were observed [37]–[39]. Furthermore, missense mutations within human *PLCz1* confer male infertility [17], [40]–[42]. This could be confirmed in an equivalent mouse model [37]–[39]. The D202R (D210R in mice) mutation of in X-catalytic domain abrogated the Ca^2+^ oscillatory ability of PLCz1 by failure of nuclear translocation [43], [44]. Recently, species-specific differences in sequence and expression patterns of equine PLCz1 were reported [35], [45]. Subfertile stallions showed a reduced PLCz1 expression [45], [46] as well as abnormal patterns of localization of PLCz1 in the sperm [45]. In pigs, higher expression levels of *PLCz1* were related with better sperm quality [47]. A positive correlation of *PLCz1* expression levels with sire conception rate was observed in Holstein bulls [48]. In Chinese Holstein bulls, semen quality traits were shown significantly associated with single SNPs in the 5′ flanking region of *PLCz1* and the haplotypes constructed from these SNPs [49].

Out of the 47 *PLCz1* polymorphisms, three SNPs (g.45595060G>T, g.45599001G>A and BIEC2-952439) reached significant associations with EBV-PAT in the validation sample after accounting for multiple testing. Using *in-silico* predictions, potential regulatory functions may be assumed for all 14 EBV-PAT-associated SNPs (Table S5). Searches for splice-modifying SNPs predicted g.45595060G>T as a candidate to disrupt the binding sites for putative exonic splicing enhancers (ESE) octamer motifs. ESEs stimulate alternative and constitutive splicing and serve as binding sites for splicing factors like the serine/arginine-rich proteins (SR proteins) [50], [51]. ESEs are exonic sequence elements. However, less frequent abundance of ESEs sequences in introns and their specific interaction with splicing factors have also been documented [51]–[53]. There are reports about deep intron mutations creating ESE motifs and splice factor binding sites which lead to pseudoexon activation [54]–[56]. Consequently, even little is known about the mechanisms by which intronic sequence variations can activate pseudoexons and may alter trait expression, we can speculate that the deep intron mutation g.45595060G>T could lead to inaccurate recognition of exon/intron boundaries and therefore modulate the splicing pattern of equine *PLCz1*.

Disruption of an intronic microRNA (miRNA) motif was predicted for the SNP g.45599001G>A. These small non-coding regulatory RNAs are able to interfere post-transcriptionally with the protein production of their targets by suppressing translation or destabilizing mRNAs [57]. They are involved in mammal core cellular processes [57], [58] as well as in many human diseases [59]–[61]. Numerous intronic sequences have been found to encode miRNAs, responsible for gene silencing [62]. Mammalian miRNAs decrease target mRNA levels and translational efficiency resulting in reduced protein output [63]. In mammalian reproduction pathways, miRNAs play a potential role in testicular and ovarian physiology [64]. MiRNAs are associated with functional regulation of Leydig cells and Sertoli cells in testis in steroid synthesis which suggest the potential involvement of miRNAs in translational control during spermatogenesis [64]. The pattern of miRNAs expressed in infertile men suggested their role in regulating male germ and somatic cells and that their alteration is associated with reproductive abnormalities [65]. A recent study in livestock animals including horses showed a profound effect of miRNA polymorphisms on a wide range of phenotypic traits in animal production [66]. The miRNA motif when removed by the SNP g.45599001G>A may alter protein expression level of PLCz1.

The SNP g.45599001G>A creates a TF for the hepatocyte nuclear factor 3-alpha (HNF-3alpha). HNF-3alpha is involved in embryonic development, establishment of tissue-specific gene expression and regulation of gene expression in differentiated tissues [67].

Comparison of the sequences generated in the present study to the annotated equine *PLCz1* sequences showed several differences. Ensembl transcript 1 (ENSECAT00000012304) and NCBI transcript (XM_001497766.3) were annotated with 13 exons whereas Ensembl transcript 2 (ENSECAT00000012372) consists of 15 exons. Non-coding exon 1 homologs were not annotated in the horse genome reference assembly EquCab2.0. The coding sequences (CDS) and protein sequences of non-PTC-containing transcripts are consistent with the Ensembl (ENSECAT00000012304 and ENSECAP00000009710) annotation, however, different to NCBI variants XM_001497766.2 and XP_001497816.2 (Figure S4). The mRNA sequences of non-PTC-containing transcripts exhibit 85% homology to human (NM_033123.3) and 75% to murine *PLCz1* (NM_054066.4) mRNA, respectively. They are predicted to encode a protein of 638 aa which displays 82% similarity to the human *PLCz1* protein (NP_149114.2) and 70% sequence similarity to the murine protein (NP_473407.2). CLUSTAL OMEGA alignment displayed high interspecies conservation across *PLCz1*, except for the X-Y linker domain.

Sequencing of *CAPZA3* cDNA revealed a transcript consistent with the annotation in Ensembl (ENSECAT00000004119). The mRNA sequence exhibits 90.3% homology to human (ENST00000317658) and 82.0% to murine *CAPZA3* (ENSMUST00000043797) mRNA, respectively. The predicted equine CAPZA3 protein displays 84.9% similarity to the human CAPZA3 protein (ENSP00000326238) and 85.2% similarity to the murine protein (ENSMUSP00000038562).

In human, two isoforms of *PLCz1* have been described. Human transcript variant 1 (NM_033123.3) encodes the functional protein whereas transcript variant 2 (NR_073075.1) is considered to be non-protein coding due to the existence of a PTC. We determined two equine transcript variants of *PLCz1* (JX545318, JX545320) that harbour a PTC at the same position as shown in human *PLCz1* transcript variant 2 (NR_073075.1). Both equine PTC-containing transcript variants are predicted to result in truncated proteins and to elicit NMD. Comparison of equine ORF2 to human *PLCz1* pinpointed a truncated protein (ENSP00000443320) with a similar size (412 aa) (Figure S4). A PTC is located >50 nucleotides upstream of the 3′most exon–exon junction [68]. PTC-containing transcripts frequently arise from alternative splicing events and elicit NMD [68], [69] in order to prevent production of potentially damaging truncated proteins [70]–[73] and to regulate normal gene expression by controlling the frequency of transcripts [74], [75]. An example for alternative splicing pattern activated by a PTC was recently described in the human *GABRG2* gene associated with childhood absence epilepsy [76], [77]. Furthermore, alternative splicing induced intron retention in human *ribosomal protein L3* (*rpL3*) gene was considered to activate PTC and targeted mRNA for NMD. Overexpression of *rpL3* caused downregulation of canonical spliced mRNA and upregulation of alternative spliced mRNA, promoted by hnRNP H1 and other splicing factors for selection of alternative splice site [78]. Therefore, we propose that the equine PTC-containing *PLCz1* transcripts may also respond to NMD.

Even if most of the eukaryotic mRNAs contain only one ORF, translation can be initiated at a downstream start codon due to leaky scanning [79], [80]. Leaky scanning skips the major ORF and enables initiation of translation of internal second ORFs [81]. A strong identity to kozak (GXXATGa) [81], [82] represented by downstream translation start of the PTC-containing transcripts may give evidence to the existence of ORF2. Nevertheless, the 534 aa protein derivated from ORF2 lacks 2/3 of N-terminal EF-hand domains (helix-loop-helix structural domains). Loss of EF-hand domains sequence is crucial for the ability of enzymes to initiate timely Ca2+ oscillation within the oocytes [35], [37], [44]. Thus, a potentially not fully functional protein will arise from ORF2. Even if all the different transcript variants could be observed in the six Hanoverian warmblood horses sequenced, it is unlikely that *PLCz1* splicing patterns share a relationship with EBV-PAT. In summary, we can hypothesize that the equine *PLCz1* transcript without a PTC may be dominantly expressed.

Even if it has been demonstrated that the closely neighbouring sperm-specific intronless *CAPZA3* is of notably interest in human [19] and mice fertility [20], [21], no significant association with EBV-PAT was found for the *CAPZA3*-related SNP in Hanoverian stallions. Based on failing validation, this gene had to be eliminated as candidate locus for Hanoverian stallions.

In conclusion, we identified *PLCz1* as an important gene for equine male fertility. The polymorphisms significantly associated with EBV-PAT may have regulatory effects on transcription levels of the gene. Several SNPs may influence the effects of *PLCz1* and these SNPs are located in three adjacent haplotype blocks.

## Materials and Methods

### Ethics statement

All animal work has been conducted according to the national and international guidelines for animal welfare. All horses included in this study were owned by the National State Stud of Lower Saxony in Celle and the Landstallmeister Dr. Brockmann of the National State Stud of Lower Saxony in Celle gave his explicit consent for this study. The Lower Saxony state veterinary office at the Niedersächsisches Landesamt für Verbraucherschutz und Lebensmittelsicherheit, Oldenburg, Germany, was the responsible Institutional Animal Care and Use Committee (IACUC) for this specific study. The EDTA-blood sampling for the present study had been approved by the IACUC of Lower Saxony, the state veterinary office Niedersächsisches Landesamt für Verbraucherschutz und Lebensmittelsicherheit, Oldenburg, Germany (registration number 33.42502-05-07A482).

When a male horse should be castrated for reasons other than research, we were informed by the National State Stud of Lower Saxony in Celle. The castration was performed by a veterinarian specialized in equine reproductive medicine (GM). Then, we could collect testis samples from these horses after castration and therefore, a specific approval through the IACUC was not necessary according to the German Animal Welfare Law (released on 05/18/2006, last changes on 07/08/2013). All testis tissue samples used in the present study were from horses that were owned by the National State Stud of Lower Saxony in Celle.

### Animals, phenotypes data, DNA and RNA extraction

EDTA-blood samples of 228 Hanoverian warmblood stallions from the National State Stud of Lower Saxony in Celle were collected for the present GWAS-study. Fertility data contained >20,000 mares with >200,000 artificial inseminations (AI) records of >600 stallions for calculation of the pregnancy rate per estrus and estimation of breeding values for the paternal component of the pregnancy rate per estrus cycle (EBV-PAT). The model used for prediction of EBV-PAT are described elsewhere [83]. Briefly, this model included an additive genetic component for the stallion and an additive genetic component for the maternal effect. Non-genetic effects taken into account included month and year of insemination, management of insemination and semen, age of the mare and number of foals given birth by the mare. Calculations were performed using an animal threshold model.

Genomic DNA from EDTA-blood samples was isolated using standard methods with red blood cell lysis buffer and sodium EDTA buffer. The DNA concentration of the samples was adjusted to 50 ng/µl using Nanodrop ND-1000 (Peqlab Biotechnology, Erlangen, Germany).

For sequence analysis of genomic DNA, we prepared genomic DNA from EDTA-blood samples of 19 Hanoverian warmblood stallions using the NucleoSplin 96 Blood DNA kit (Macherey-Nagel, Düren, Germany) according to the manufacturer's instructions. This sample contained 19 fertile Hanoverian stallions with low, moderate, high or very high EBV-PAT (Table S1). These 19 stallions were not contained in the detection and validation sample.

Testis tissue samples were collected from six male Hanoverian horses that were castrated due to the owner's decision. For sequencing cDNA, testis tissue was conserved in RNAlater solution (Qiagen, Hilden, Germany) or liquid nitrogen. The RNeasy 96 Lipid Tissue Kit (Qiagen) was used to extract total RNA from testis tissue. The RNA was transcribed into cDNA using Maxima First Strand cDNA Synthesis Kit for RT-qPCR (Fermentas, St. Leon-Rot, Germany) according to the manufacturer's protocol.

For validation of candidate gene-associated polymorphisms, genomic DNA samples of 237 Hanoverian stallions were analysed. This sample included the 228 Hanoverian stallions employed for the GWAS.

### Genome-wide association study

A total of 228 Hanoverian stallions were genotyped using the Equine SNP50 Beadchip (Illumina, San Diego, CA, USA) including 54,602 SNPs. Quality criteria were minor allele frequency (MAF) >0.05, genotyping rate per SNP and animal >0.90 and HWE (P<0.00001). After filtering for quality criteria, 46,074 SNPs remained for the final analysis. A mixed linear model (MLM) was employed to control spurious associations caused by population structure. The MLM included genotype as fixed effect and a random animal effect through an identity-by-state-kinship (IBS) matrix. A Q-matrix to detect population structure was estimated using principal components (PCAs). We tested several extended models employing up to six PCAs to show the robustness of the outcome of the GWAS. All these models yielded the same associated SNPs as the final model. Thus, adding principal components for cryptic data structure did not change the results of the final model. Thus, the IBS matrix reflected the genomic relationship matrix among all individuals genotyped and captured the relatedness among animals as well as the cryptic family structure. The analysis was run using TASSEL, version 3.0.146 [85]. The adaptive false discovery rate according to Benjamini-Hochberg and the Bonferroni correction were calculated using the MULTIPLE TEST procedure of SAS, version 9.3, to determine the threshold for experiment-wide significance. The haplotype structure of the associated genomic regions was analyzed using Haploview 4.2 [23].

### Bioinformatic analyses and sequencing

In order to build an equine gene model, we employed human mRNA as a reference sequence. Using BLAST (http://www.ncbi.nlm.nih.gov/BLAST/) and SPIDEY (http://www.ncbi.nlm.nih.gov/spidey/), we determined the equine sequence and its genomic location on equine chromosome 6. To compare horse, human and murine mRNAs and protein sequences we used the multiple-alignment program CLUSTALW2 (http://www.ebi.ac.uk/Tools/msa/clustalw2/). The splice site prediction tools Human Splicing Finder (HSF) 2.4.1 (www.umd.be/HSF/) and NetGene2 (www.cbs.dtu.dk/services/NetGene2) were used to predict the effect of intronic variants on splicing processes and to analyze the effect of polymorphisms on putative splicing regulatory sequences. Further, we used HSF 2.4.1 sequence analysis matrices to identify branch point, enhancer and silencer motifs. SNPs influencing transcription factor binding sites were predicted using Alibaba 2.1 (www.gene-regulation.com/pub/programs/alibaba2/bin/index.html) and Patch1.0 (www.gene-regulation.com/cgi-bin/pub/programs/patch/bin/patch.cgi). To predict potentially regulatory micro RNA (miRNA) motifs in human and equine sequences with essential roles in transcriptional and post-transcriptional regulation of gene expression, we employed miRBase, a miRNA database (http://www.mirbase.org/) [84], [85]. The software PolyPhen-2 (http://genetics.bwh.harvard.edu/pph2/) [22] was used to predict possible impact of an amino acid substitution on the structure and function of the equine protein. Open reading frame prediction was performed using ORF finder (http://www.ncbi.nlm.nih.gov/projects/gorf/), prediction of initiation codons in cDNA sequences was performed using ATG_PR_ (http://atgpr.dbcls.jp/).

The genes *PLCz1* and *CAPZA3* were selected for sequencing using genomic DNA and cDNA. We amplified all exons, exon-intron boundaries and the complete intron 8 of *PLCz1* containing the EBV-PAT-associated SNP BIEC2-952439 (Table S6). The primer sequences were obtained using equine reference mRNA and genomic sequence of ECA6 (ENSECAT00000012304 and ENSECAG00000011373) of the horse genome reference assembly EquCab2.0. The primer design was carried out with Primer3 (http://bioinfo.ut.ee/primer3-0.4.0/) after masking repetitive sequences using the RepeatMasker (http://www.repeatmasker.org/). In order to detect variation in non-annotated equine exon 1 of *PLCz1*, we designed several 5′UTR primer combinations as shown in Table S6. PCR reactions were performed in 20-µl reaction volumes using 10 ng DNA, 100 µM dNTPS, 10 pmol of each primer and 2 units/µl *Taq* DNA polymerase (MP Biomedicals, Eschwege). After 4 min initial denaturation at 94°C, 35 cycles of 30 s at 94°C, 30 s annealing temperature and 45 s at 72°C and 10 min final extension at 72°C were performed on a PTC 100 thermal cyclers (MJ Research, Watertown, MA, USA). After purification the PCR products using EXO-SAP, amplicons were directly sequenced using the Big Dye Sequencing Kit (Life Technologies, Darmstadt, Germany) on an ABI Prism 3500 (Life Technologies).

### Mutation analysis and genotyping of polymorphisms in the validation sample

Sequence data were analysed using Sequencher 4.8. (Gene Code, Ann Arbor, MI, USA). All 48 polymorphisms identified were tested for association in the detection sample (n = 19) and validated in 237 Hanoverian stallions. Genotyping of polymorphisms was performed using PCR-RFLP or TaqMan SNP Genotyping Assay (Table S7). For enzymatic digestion, we used 15-µl reaction volume containing 1.5 µl buffer, 1.5 U endonucleases and 5 µl PCR amplicons. A mismatch PCR-RFLP technique [86] was used in the case that no restriction site was present. For genotyping the exonic SNP g.45599207G>A, a custom TaqMan SNP Genotyping Assay (Life Technologies) was applied. The assay incorporates 5′ allele-specific labeled TaqMan MGB probes (FAM and VIC dye-labeled) and unlabeled assay primers. The assay contained in 12-µl reaction volume Maxima Probe qPCR Master Mix 2× (Fermentas, St. Leon-Rot), 0.07 ROX solution 50 µM (Fermentas, St. Leon-Rot), 0.25 µl Custom TaqMan SNP genotyping assay and 2 µl template DNA. RT-PCR was carried out using the Applied Biosystems 7300 Real-Time PCR System (Life Technologies). The indel mutation g.45581388delTTAA was genotyped using DY-682-tagged primers (Eurofins MWG Operon, Ebersberg, Germany). PCR reactions were performed as described above. The PCR products were analyzed by gel electrophoresis on the automated sequencer LI-COR 4300 (LI-COR, Bad Homburg, Germany) using a 4% polyacrylamide denaturing gels (Rotiphorese Gel40, Carl Roth, Karlsruhe, Germany). Differences in allele sizes caused by deleted or inserted bases were defined by visual examination.

### Statistical analysis of the validation samples

The ALLELE procedure of SAS/Genetics was used to calculate allele and genotype frequencies, the minor allele frequency (MAF), the polymorphism information content (PIC), the observed heterozygosity (HET), allelic diversity and χ^2^-tests for Hardy-Weinberg equilibrium for the SNPs genotyped in the validation sample. Calculation of association was performed using PLINK, version 1.07, and the procedure GLM of SAS, version 9.3. Correction for multiple testing according to Bonferroni was done using the MULTIPLE TEST procedure of SAS, version 9.3. Imputation of genotypes was carried out using BEAGLE 3.3.2 [87], [88]. We applied a population linkage disequilibrium based localized haplotype clustering method. We selected all polymorphisms sequenced (n = 48) in 19 stallion and a subset of 16 SNPs genotyped in 237 animals as reference cohort to impute the remaining genetic variants within *PLCz1*.

Calculation of pairwise linkage disequilibria (LD) was performed and visualized using Haploview 4.2 [23]. To define haplotype blocks and to analyse the haplotype structure of the *PLCz1-*associated polymorphisms, we used the four gamete rule algorithm with a D' value >0.8. We excluded polymorphisms with a MAF<0.01. The proportion of variance explained for EBV-PAT by polymorphisms or haplotypes was estimated using the procedure GLM of SAS, version 9.3.

## Supporting Information

Figure S1 **Haplotype structure of the genomic region at 44,000,970–46,952,651 base pairs on horse chromosome (ECA) 6 and genes annotated on the horse genome reference assembly EquCab2.0.** Protein-coding genes are highlighted in bold, pseudogenes are marked in grey and non-coding RNAs are marked in light purple. Haplotype blocks were defined using the four gamete rule algorithm. The haplotype block 9 in intron 8 of the equine *PLCz1* contains the SNP BIEC2-952439 significantly associated with estimated breeding values of the paternal component of the pregnancy rate per estrus cycle (EBV-PAT) in Hanoverian stallions. The figure displays Hendrige's multiallelic D, which represent the degree of linkage disequilibrium between each two SNPs. Red fields display LOD≥2 (D′ = 1), shades of red show the same LOD with D′<1. White and blue fields display LOD<2 with D′<1 and D′ = 1, respectively.
(TIF)

Figure S2 **Transcript variants of equine** ***PLCz1*** **detected.** (A) Usage of variant-specific 5′ UTR primer revealed non-coding exon 1 variation shown in transcripts. Blue errors indicate position of specific primers and evaluated start for exon 1a and exon 1b. Exons show overlapping position, with an exon 1b position more downstream of exon 1a. Due to study design, definitely 5′ start of exons 1 was not determined, indicated by dashed lines. (B) Comparison of mature mRNA transcript variants. They differ in size due to usage of variable exon1 (1a and 1b) and exon 4 (4a and 4b).
(TIF)

Figure S3 **Transcript variants of equine** ***PLCz1*** **detected in testis tissue.** Sequence analyses revealed four different transcripts variants. Translation start and stop are given for each transcript (A: JX545319, B: JX545320, C: JX545317, D: JX545318). Variants show different size of non-coding exon 1. Exon 1a represented in the first primary transcript (A) and in derived transcript (B) contains 114 base pairs (bp), whereas exon 1b represented in second primary transcript (C) and in derived transcript (D) consists of 174 bp. The non- premature-terminating-codon (PTC) containing variants (A, C) consist of the translated exon 4a whereas truncated variants (B, D) show a PTC in exon 4b leading to two potential open reading frames (ORFs, ORF1 and ORF2). Translated exons are shown as solid black boxes. Untranslated exon regions are shown as open boxes. The scale is in bp.
(TIF)

Figure S4 **Protein sequence alignments of equine and human PLCz1 variants.** (1,2) Sequences highlighted in yellow list truncated PLCz1 protein of 45 amino acids (aa) in human (ENSP00000326397) and equine (JX545318, JX545320). (3–6) Sequence variations of predicted equine protein annotation (3: JX545317, JX545319; 4: ENSECAP00000009710; 5: ENSECAP00000009768; 6: XP_001497816.2) are shown. (7) Interspecies alignment with reviewed human peptide sequence of PLCz1 (NP_149114.2). (8,9) Sequences highlighted in green list truncated PLCz1 protein of 412 aa and 534 aa in human (ENSP00000443320) and equine (JX545318, JX545320). Truncated sequences may be generated due to activation of the premature termination codon (PTC) or are thought to undergo nonsense mediated decay (NMD). The open boxed region corresponds to the N-terminus located EF-hand domain, whereas the boxed region marks the X-Y linker domain. Human sequences are highlighted in bold.
(TIF)

Figure S5 **Linkage disequilibria (LD) among 47 polymorphisms within** ***PLCz1*** **in 237 Hanoverian stallions.** (A) The LD display shows Hedrige's multiallelic D which represents the degree of LD between two blocks. Red fields display LOD≥2 (D′ = 1), shades of red show the same LOD with D′<1. White and blue fields display LOD<2 with D′<1 and D′ = 1, respectively. The pairwise LD coefficients (r^2^) values are shown for each SNP pair in fields. (B) Haplotype blocks were defined using the four gamete rule algorithm. Each haplotype in a block with its frequency and connections from one block to the next block is displayed. In the crossing areas, a value for the multiallelic D′ is shown, representing the level of recombination between the two blocks. Significantly associated haplotype blocks and significantly associated SNPs within haplotype blocks are framed in red.
(TIF)

Table S1 **Hanoverian stallions used in the detection sample and their estimated breeding values of the paternal component of the pregnancy rate per estrus cycle (EBV-PAT) on the natural scale and standardized scale (R-EBV-PAT: 100±20) as well as the accuracy of EBV-PAT.** Stallions were sorted in four groups according to their EBV-PAT.
(DOCX)

Table S2 **Polymorphisms identified in equine** ***PLCz1*** **and** ***CAPZA3*** **.** Identification of polymorphisms, their accession numbers, their intragenic locations and their possible effects on the coding sequence including the nomenclature for the protein changing mutations are given.
(DOCX)

Table S3 **Association analysis for 48 polymorphisms with the estimated breeding values of the paternal component of the pregnancy rate per estrus cycle (EBV-PAT) in the detection sample using 19 Hanoverian stallions.** The variance explained (R^2^) by each polymorphism, the P-values (P) for association with EBV-PAT and minor allele frequencies (MAF) are given.
(DOCX)

Table S4 **Haplotypes within** ***PLCz1*** **significantly associated with the estimated breeding values of the paternal component of the pregnancy rate per estrus cycle (EBV-PAT) in 237 Hanoverian stallions.** Significant haplotypes, their frequencies, corresponding haplotype blocks, least-square means (LSM) with their standard errors (SE) and P-values (P) are given.
(DOCX)

Table S5 **Predicted influence of EBV-PAT-associated polymorphisms within** ***PLCz1*** **on transcription factor (TF) binding sites, microRNA (miRNA) and splice-site modifying motifs.**
(DOCX)

Table S6 **Primer sequences and their positions, product sizes and annealing temperatures (AT) for sequencing the equine genomic DNA and cDNA of** ***PLCz1*** **and** ***CAPZA3*** **.**
(DOCX)

Table S7 **Genotyping techniques used for validation of polymorphisms in 237 Hanoverian stallions.** All single nucleotide polymorphisms (SNPs) genotyped using RFLPs (restriction fragment length polymorphisms) or IRD (infrared dye) labeled gel electrophoresis are given.
(DOCX)
