# Supplementary figures and images for: Genome-Wide Association Study Identifies Phospholipase C zeta 1 (PLCz1) as a Stallion Fertility Locus in Hanoverian Warmblood Horses
Source: PLoS One. 2014 Oct 29;9(10):e109675. doi: 10.1371/journal.pone.0109675 (PMC4212906; doi:10.1371/journal.pone.0109675)

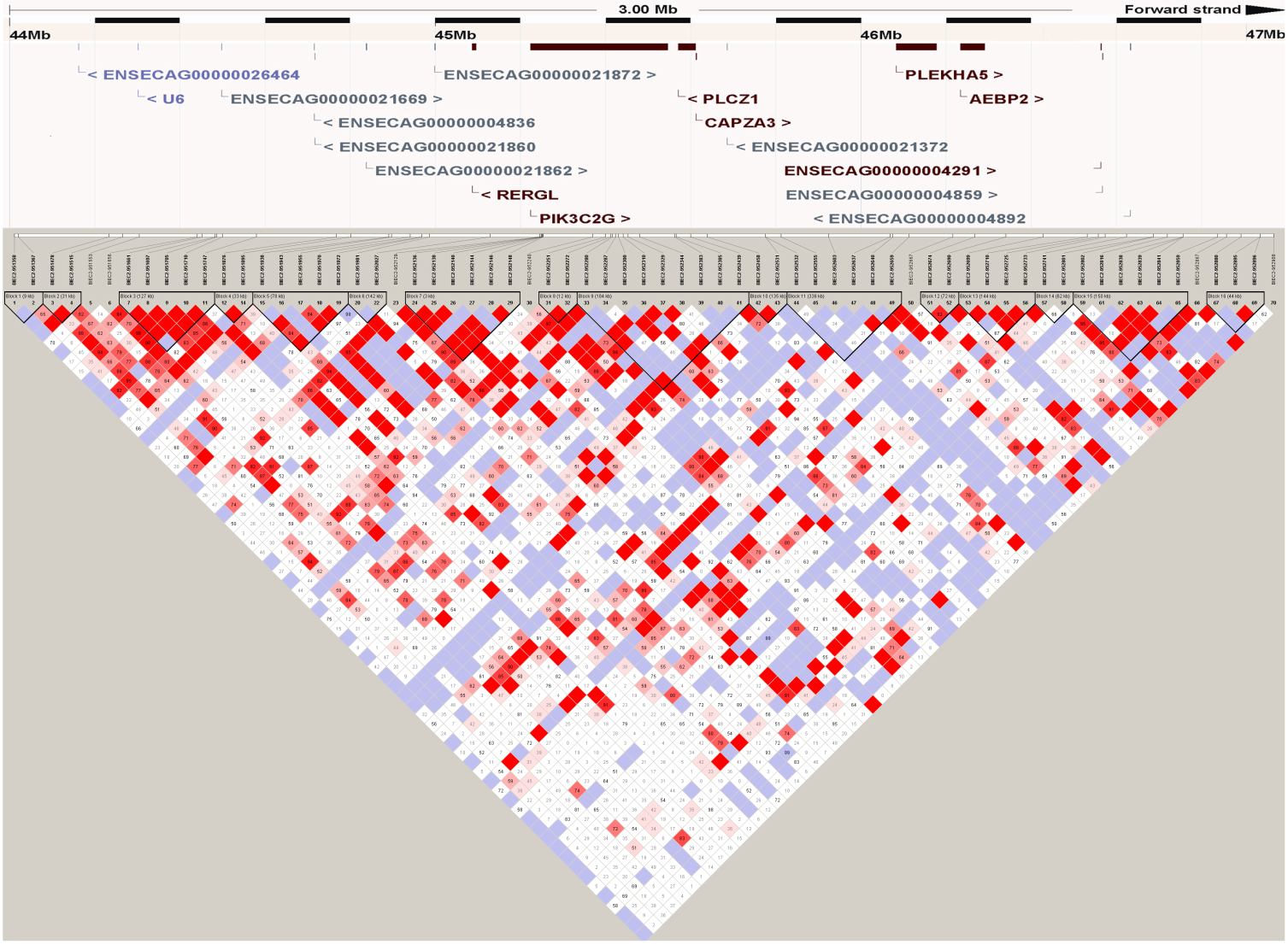

Supplement: Figure S1 — Haplotype structure of the genomic region at 44,000,970–46,952,651 base pairs on horse chromosome (ECA) 6 and genes annotated on the horse genome reference assembly EquCab2.0. Protein-coding genes are highlighted in bold, pseudogenes are marked in grey and non-coding RNAs are marked in light purple. Haplotype blocks were defined using the four gamete rule algorithm. The haplotype block 9 in intron 8 of the equine PLCz1 contains the SNP BIEC2-952439 significantly associated with estimated breeding values of the paternal component of the pregnancy rate per estrus cycle (EBV-PAT) in Hanoverian stallions. The figure displays Hendrige's multiallelic D, which represent the degree of linkage disequilibrium between each two SNPs. Red fields display LOD≥2 (D′ = 1), shades of red show the same LOD with D′<1. White and blue fields display LOD<2 with D′<1 and D′ = 1, respectively. (TIF) [file pone.0109675.s001.tif]

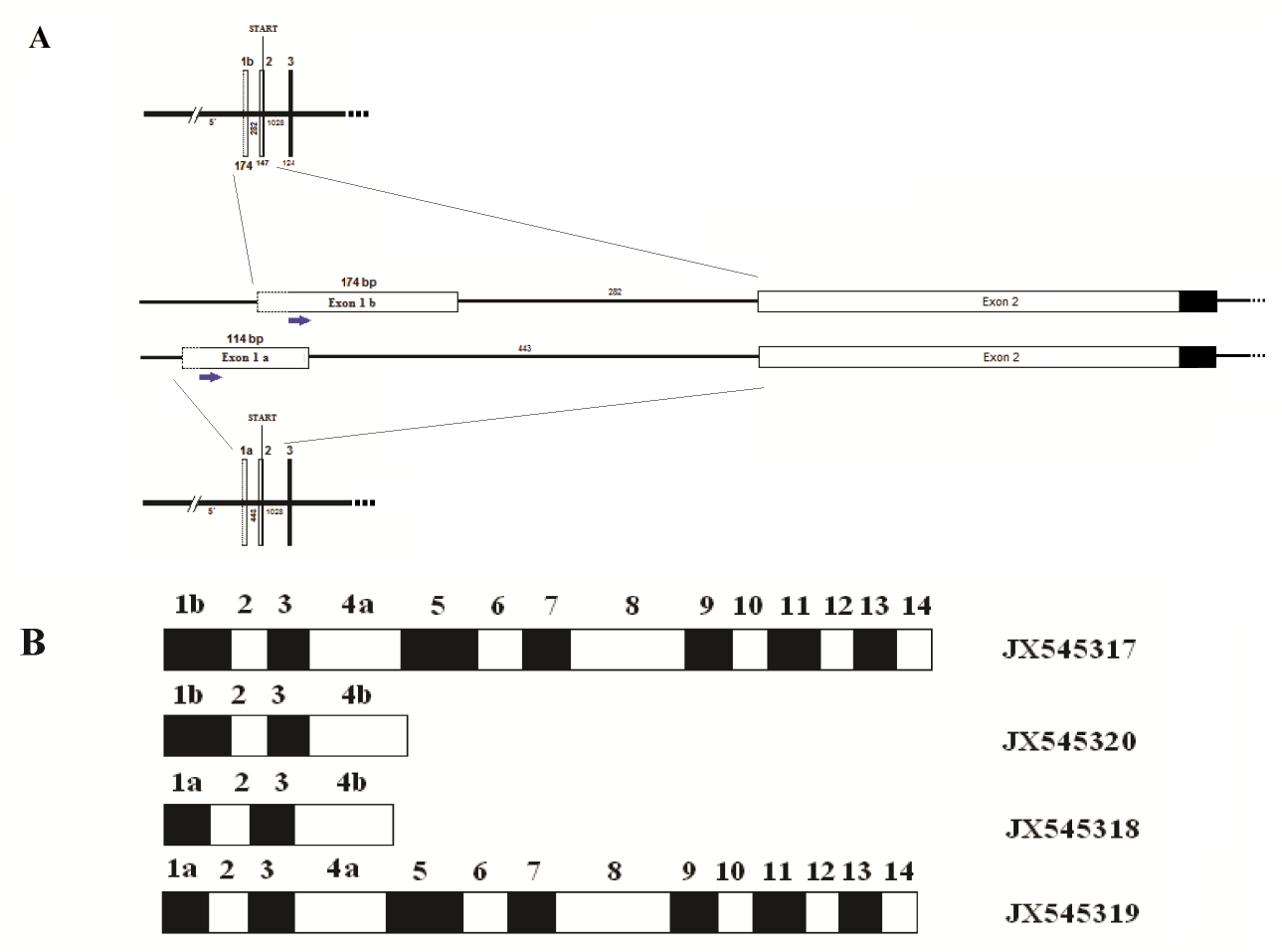

Supplement: Figure S2 — Transcript variants of equine PLCz1 detected. (A) Usage of variant-specific 5′ UTR primer revealed non-coding exon 1 variation shown in transcripts. Blue errors indicate position of specific primers and evaluated start for exon 1a and exon 1b. Exons show overlapping position, with an exon 1b position more downstream of exon 1a. Due to study design, definitely 5′ start of exons 1 was not determined, indicated by dashed lines. (B) Comparison of mature mRNA transcript variants. They differ in size due to usage of variable exon1 (1a and 1b) and exon 4 (4a and 4b). (TIF) [file pone.0109675.s002.tif]

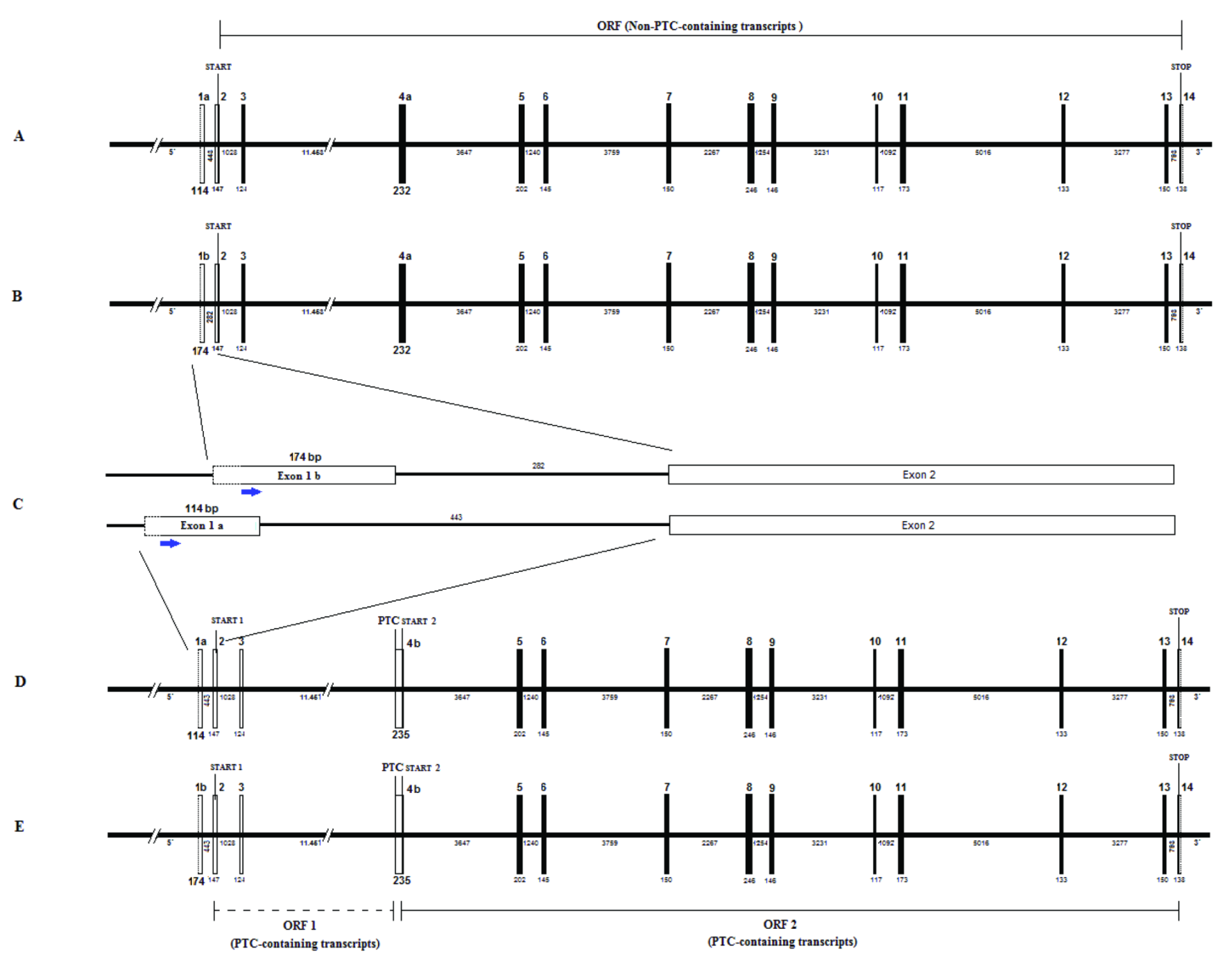

Supplement: Figure S3 — Transcript variants of equine PLCz1 detected in testis tissue. Sequence analyses revealed four different transcripts variants. Translation start and stop are given for each transcript (A: JX545319, B: JX545320, C: JX545317, D: JX545318). Variants show different size of non-coding exon 1. Exon 1a represented in the first primary transcript (A) and in derived transcript (B) contains 114 base pairs (bp), whereas exon 1b represented in second primary transcript (C) and in derived transcript (D) consists of 174 bp. The non- premature-terminating-codon (PTC) containing variants (A, C) consist of the translated exon 4a whereas truncated variants (B, D) show a PTC in exon 4b leading to two potential open reading frames (ORFs, ORF1 and ORF2). Translated exons are shown as solid black boxes. Untranslated exon regions are shown as open boxes. The scale is in bp. (TIF) [file pone.0109675.s003.tif]

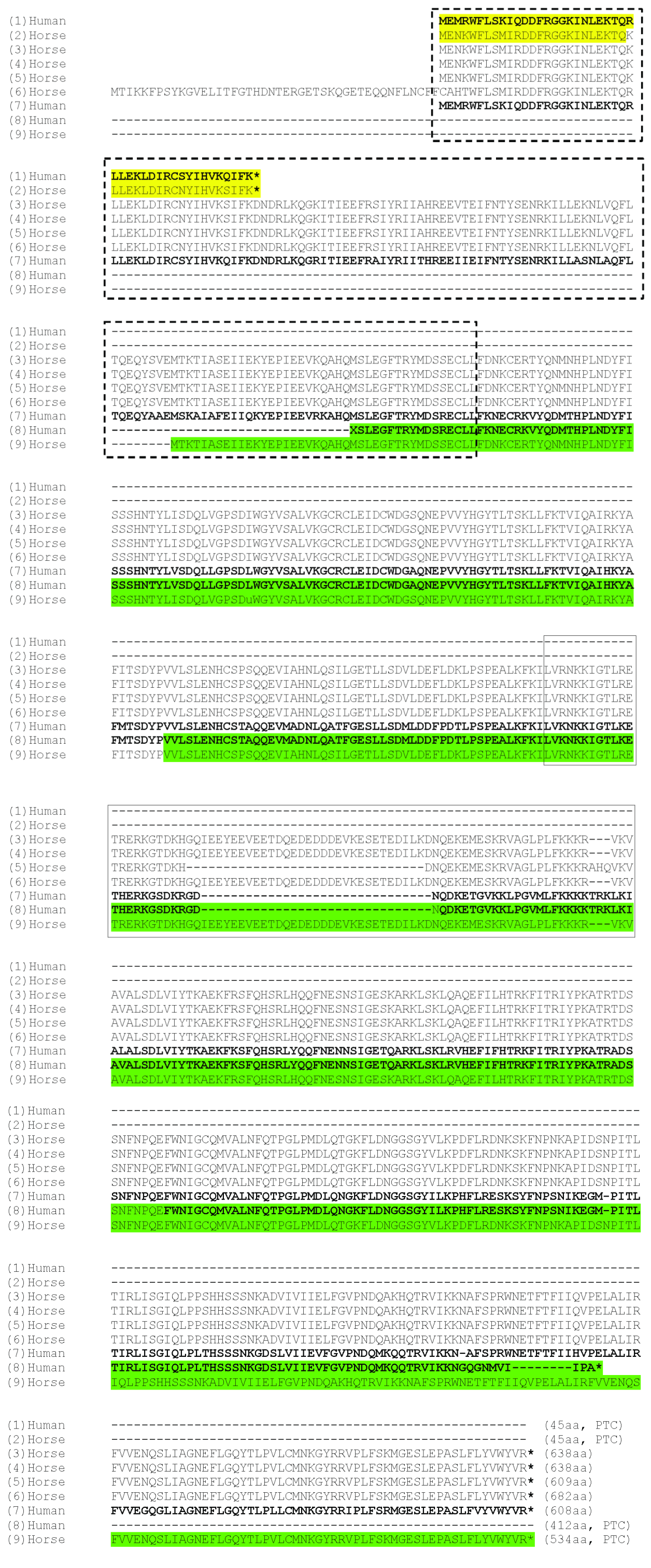

Supplement: Figure S4 — Protein sequence alignments of equine and human PLCz1 variants. (1,2) Sequences highlighted in yellow list truncated PLCz1 protein of 45 amino acids (aa) in human (ENSP00000326397) and equine (JX545318, JX545320). (3–6) Sequence variations of predicted equine protein annotation (3: JX545317, JX545319; 4: ENSECAP00000009710; 5: ENSECAP00000009768; 6: XP_001497816.2) are shown. (7) Interspecies alignment with reviewed human peptide sequence of PLCz1 (NP_149114.2). (8,9) Sequences highlighted in green list truncated PLCz1 protein of 412 aa and 534 aa in human (ENSP00000443320) and equine (JX545318, JX545320). Truncated sequences may be generated due to activation of the premature termination codon (PTC) or are thought to undergo nonsense mediated decay (NMD). The open boxed region corresponds to the N-terminus located EF-hand domain, whereas the boxed region marks the X-Y linker domain. Human sequences are highlighted in bold. (TIF) [file pone.0109675.s004.tif]

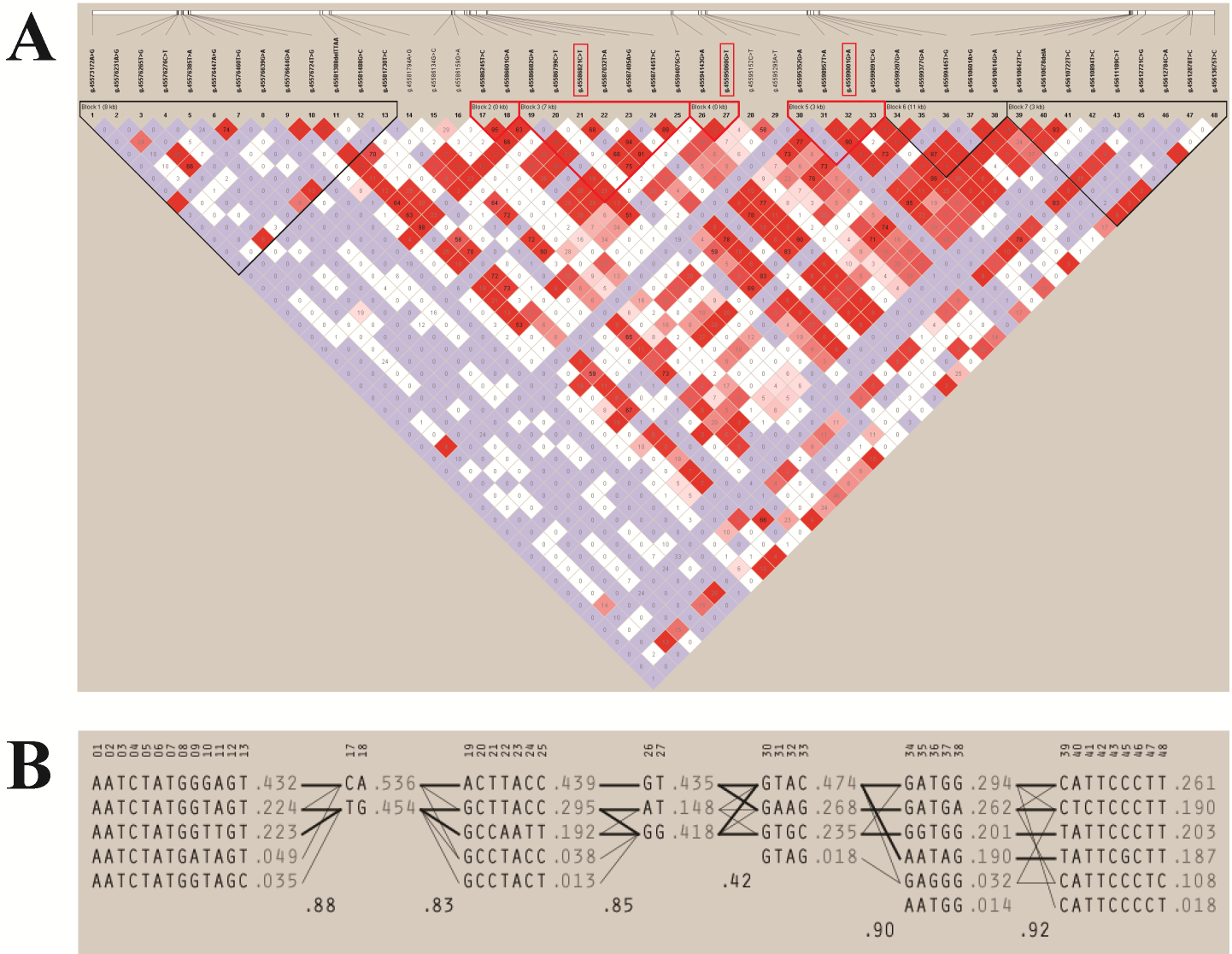

Supplement: Figure S5 — Linkage disequilibria (LD) among 47 polymorphisms within PLCz1 in 237 Hanoverian stallions. (A) The LD display shows Hedrige's multiallelic D which represents the degree of LD between two blocks. Red fields display LOD≥2 (D′ = 1), shades of red show the same LOD with D′<1. White and blue fields display LOD<2 with D′<1 and D′ = 1, respectively. The pairwise LD coefficients (r2) values are shown for each SNP pair in fields. (B) Haplotype blocks were defined using the four gamete rule algorithm. Each haplotype in a block with its frequency and connections from one block to the next block is displayed. In the crossing areas, a value for the multiallelic D′ is shown, representing the level of recombination between the two blocks. Significantly associated haplotype blocks and significantly associated SNPs within haplotype blocks are framed in red. (TIF) [file pone.0109675.s005.tif]
