# Supplementary material for: Genome-Wide Association Study Identifies Phospholipase C zeta 1 (PLCz1) as a Stallion Fertility Locus in Hanoverian Warmblood Horses
Source: PLoS One. 2014 Oct 29;9(10):e109675. doi: 10.1371/journal.pone.0109675 (PMC4212906; doi:10.1371/journal.pone.0109675)
Supplement: Table S1 — Hanoverian stallions used in the detection sample and their estimated breeding values of the paternal component of the pregnancy rate per estrus cycle (EBV-PAT) on the natural scale and standardized scale (R-EBV-PAT: 100±20) as well as the accuracy of EBV-PAT. Stallions were sorted in four groups according to their EBV-PAT. (DOCX) [file pone.0109675.s006.docx]

**Table S1. Hanoverian stallions used in the detection sample and their estimated breeding values of the paternal component of the pregnancy rate per estrus cycle (EBV-PAT) on the natural scale and standardized scale (R-EBV-PAT: 100 ± 20) as well as the accuracy of EBV-PAT.** Stallions were sorted in four groups according to their EBV-PAT.

| EBV-PAT | Stallion | EBV-PAT | r_AI_ | R-EBV-PAT |
| --- | --- | --- | --- | --- |
| Group | ID |  |  |  |
| Very low |  |  |  |  |
|  | 1 | -0.2963 | 0.77 | 21 |
|  | 2 | -0.2233 | 0.70 | 40 |
|  | 3 | -0.1905 | 0.70 | 49 |
|  | 4 | -0.1787 | 0.74 | 52 |
|  | 5 | -0.1139 | 0.70 | 69 |
|  | 6 | -0.0707 | 0.70 | 80 |
| Moderate |  |  |  |  |
|  | 7 | -0.0380 | 0.70 | 89 |
|  | 8 | -0.0244 | 0.70 | 92 |
|  | 9 | -0.0192 | 0.70 | 94 |
|  | 10 | -0.0057 | 0.70 | 97 |
|  | 11 | -0.0016 | 0.71 | 98 |
|  | 12 | -0.0006 | 0.71 | 98 |
| High |  |  |  |  |
|  | 13 | 0.0203 | 0.70 | 104 |
|  | 14 | 0.0245 | 0.70 | 105 |
|  | 15 | 0.0256 | 0.74 | 106 |
| Very high |  |  |  |  |
|  | 16 | 0.0529 | 0.72 | 113 |
|  | 17 | 0.1033 | 0.70 | 126 |
|  | 18 | 0.1527 | 0.73 | 139 |
|  | 19 | 0.1699 | 0.70 | 143 |
