# Supplementary material for: Genome-Wide Association Study Identifies Phospholipase C zeta 1 (PLCz1) as a Stallion Fertility Locus in Hanoverian Warmblood Horses
Source: PLoS One. 2014 Oct 29;9(10):e109675. doi: 10.1371/journal.pone.0109675 (PMC4212906; doi:10.1371/journal.pone.0109675)
Supplement: Table S2 — Polymorphisms identified in equine PLCz1 and CAPZA3 . Identification of polymorphisms, their accession numbers, their intragenic locations and their possible effects on the coding sequence including the nomenclature for the protein changing mutations are given. (DOCX) [file pone.0109675.s007.docx]

**Table S2. Polymorphisms identified in equine *PLCz1* and *CAPZA3.*** Identification of polymorphisms, their accession number, localisation within gene and effects on the coding sequence including the nomenclature for the protein changing mutations are given.

| Polymorphism ID | Accession no. | Gene | Location | Coding sequence |
| --- | --- | --- | --- | --- |
|  |  |  |  | mutations |
| g.45613675T>C | ss748770496 | *CAPZA3* | Exon 1 | p.(Leu259Pro) |
| g.45612878T>C | ss538789602 | *PLCz1* | 5’promotor region |  |
| g.45612784C>A | ss538789603 | *PLCz1* | 5’promotor region |  |
| g.45612721C>G | ss538789604 | *PLCz1* | 5’promotor region |  |
| g.45612076G>A | ss748775027 | *PLCz1* | 5’UTR |  |
| g.45611189C>T | ss538789640 | *PLCz1* | Intron 2 |  |
| g.45610894T>C | ss784304468 | *PLCz1* | Exon 3 | p.(Asn20Ser) |
| g.45610722T>C | ss538789641 | *PLCz1* | Intron 3 |  |
| g.45610678delA | ss748770494 | *PLCz1* | Intron 3 |  |
| g.45610642T>C | ss538789643 | *PLCz1* | Intron 3 |  |
| g.45610614G>A | ss538789644 | *PLCz1* | Intron 3 |  |
| g.45610601A>G | ss538789645 | *PLCz1* | Intron 3 |  |
| g.45599445T>G | ss538789646 | *PLCz1* | Intron 3 |  |
| g.45599377G>A | BIEC2-1001104 | *PLCz1* | Intron 3 |  |
| g.45599207G>A | ss538789612 | *PLCz1* | Exon 4 | p.(Ile97Thr) |
| g.45599091C>G | ss538955058 | *PLCz1* | Intron 4 |  |
| g.45599001G>A | ss538789648 | *PLCz1* | Intron 4 |  |
| g.45598957T>A | ss538789649 | *PLCz1* | Intron 4 |  |
| g.45595352A>G | ss538789616 | *PLCz1* | Exon 5 | synonymous |
| g.45595295A>T | ss538789617 | *PLCz1* | Exon 5 | synonymous |
| g.45595152C>T | ss538789618 | *PLCz1* | Intron 5 |  |
| g.45595060G>T | ss538789654 | *PLCz1* | Intron 5 |  |
| g.45594143G>A | ss538789620 | *PLCz1* | Intron 5 |  |
| g.45594075C>T | ss538789621 | *PLCz1* | Intron 5 |  |
| g.45587445T>C | ss538789657 | *PLCz1* | Intron 8 |  |
| g.45587405A>G | ss538789658 | *PLCz1* | Intron 8 |  |
| g.45587032T>A | ss538789659 | *PLCz1* | Intron 8 |  |
| g.45586821C>T | BIEC2-952439 | *PLCz1* | Intron 8 |  |
| g.45586799C>C | ss538789625 | *PLCz1* | Intron 8 |  |

**Table S2 continued.**

| Polymorphism ID | Accession no. | Gene | Location | Coding sequence |
| --- | --- | --- | --- | --- |
|  |  |  |  | mutations |
| g.45586682G>A | ss538789626 | *PLCz1* | Intron 8 |  |
| g.45586601G>A | BIEC2-1001098 | *PLCz1* | Intron 8 |  |
| g.45586245T>C | ss538789662 | *PLCz1* | Intron 8 |  |
| g.45586159G>A | ss538955074 | *PLCz1* | Exon 9 | synonymous |
| g.45586134G>C | ss748770491 | *PLCz1* | Exon 9 | p.(Gln400Glu) |
| g.45581794A>G | BIEC2-1001094 | *PLCz1* | Intron 10 |  |
| g.45581730T>C | ss538955075 | *PLCz1* | Intron 10 |  |
| g.45581488G>C | ss784304472 | *PLCz1* | Exon 11 | p.(Asp507Glu) |
| g.45581388delTTAA | ss784304473 | *PLCz1* | Intron 11 |  |
| g.45576724T>G | ss538789663 | *PLCz1* | Intron 11 |  |
| g.45576644G>A | BIEC2-1001092 | *PLCz1* | Intron 11 |  |
| g.45576639G>A | ss538789664 | *PLCz1* | Intron 11 |  |
| g.45576460T>G | ss538789630 | *PLCz1* | Intron 11 |  |
| g.45576447A>G | ss538789631 | *PLCz1* | Intron 11 |  |
| g.45576385T>A | ss538789632 | *PLCz1* | Exon 12 | synonymous |
| g.45576276C>T | ss538955081 | *PLCz1* | Intron 12 |  |
| g.45576265T>G | ss538789669 | *PLCz1* | Intron 12 |  |
| g.45576231A>G | ss538789670 | *PLCz1* | Intron 12 |  |
| g.45573172A>G | ss538789636 | *PLCz1* | Intron 12 |  |
