# Supplementary material for: Genome-Wide Association Study Identifies Phospholipase C zeta 1 (PLCz1) as a Stallion Fertility Locus in Hanoverian Warmblood Horses
Source: PLoS One. 2014 Oct 29;9(10):e109675. doi: 10.1371/journal.pone.0109675 (PMC4212906; doi:10.1371/journal.pone.0109675)
Supplement: Table S3 — Association analysis for 48 polymorphisms with the estimated breeding values of the paternal component of the pregnancy rate per estrus cycle (EBV-PAT) in the detection sample using 19 Hanoverian stallions. The variance explained (R2) by each polymorphism, the P-values (P) for association with EBV-PAT and minor allele frequencies (MAF) are given. (DOCX) [file pone.0109675.s008.docx]

**Table S3. Association analysis for 48 polymorphisms with the estimated breeding values of the paternal component of the pregnancy rate per estrus cycle (EBV-PAT) in the detection sample using 19 Hanoverian stallions.** The variance explained (R^2^) by each polymorphism, the P-values (P) for association with EBV-PAT and minor allele frequencies (MAF) are given.

| SNP ID | Gene | Location | P | R^2^ | MAF |
| --- | --- | --- | --- | --- | --- |
| g.45612721C>G | *PLCz1* | 5‘ promotor | 0.0147 | 0.3024 | 0.16 |
| g.45595152C>T | *PLCz1* | Intron 5 | 0.0170 | 0.2917 | 0.21 |
| g.45599207G>A | *PLCz1* | Exon 4 | 0.0182 | 0.2866 | 0.24 |
| g.45610678delA | *PLCz1* | Intron 3 | 0.0224 | 0.2707 | 0.21 |
| g.45599091C>G | *PLCz1* | Intron 4 | 0.0357 | 0.2345 | 0.26 |
| g.45594143G>A | *PLCz1* | Intron 5 | 0.0486 | 0.2098 | 0.05 |
| g.45586821C>T | *PLCz1* | Intron 8 | 0.0525 | 0.2037 | 0.37 |
