# Supplementary material for: Genome-Wide Association Study Identifies Phospholipase C zeta 1 (PLCz1) as a Stallion Fertility Locus in Hanoverian Warmblood Horses
Source: PLoS One. 2014 Oct 29;9(10):e109675. doi: 10.1371/journal.pone.0109675 (PMC4212906; doi:10.1371/journal.pone.0109675)
Supplement: Table S4 — Haplotypes within PLCz1 significantly associated with the estimated breeding values of the paternal component of the pregnancy rate per estrus cycle (EBV-PAT) in 237 Hanoverian stallions. Significant haplotypes, their frequencies, corresponding haplotype blocks, least-square means (LSM) with their standard errors (SE) and P-values (P) are given. (DOCX) [file pone.0109675.s009.docx]

**Table S4. Haplotypes within *PLCz1* significantly associated with the estimated breeding values of the paternal component of the pregnancy rate per estrus cycle (EBV-PAT) in 237 Hanoverian stallions.** Significant haplotypes, their frequencies, corresponding haplotype blocks, least-square means (LSM) with their standard errors (SE) and P-values (P) are given.

| Haplotype block | Haplotype | P | LSM _EBV-PAT_ | SE |
| --- | --- | --- | --- | --- |
| Block 2 | CA | 0.0005 | 0.0373 | 0.01 |
| Block 3 | ACTTACC | 0.0006 | 0.0282 | 0.01 |
|  | GCCTACT | 0.0004 | -0.2051 | 0.06 |
| Block 4 | AT | 0.0085 | 0.0354 | 0.01 |
|  | GT | 0.0098 | 0.0328 | 0.01 |
| Block 5 | AAAG | 0.0436 | 0.170 | 0.08 |
|  | GAAG | 0.0260 | 0.023 | 0.01 |
|  | GTAC | 0.0036 | 0.035 | 0.01 |
