# Supplementary material for: Genome-Wide Association Study Identifies Phospholipase C zeta 1 (PLCz1) as a Stallion Fertility Locus in Hanoverian Warmblood Horses
Source: PLoS One. 2014 Oct 29;9(10):e109675. doi: 10.1371/journal.pone.0109675 (PMC4212906; doi:10.1371/journal.pone.0109675)
Supplement: Table S5 — Predicted influence of EBV-PAT-associated polymorphisms within PLCz1 on transcription factor (TF) binding sites, microRNA (miRNA) and splice-site modifying motifs. (DOCX) [file pone.0109675.s010.docx]

| SNP | Position in gene | TF binding site | miRNA motif | Splice-modifying motif |
| --- | --- | --- | --- | --- |
| g.45595060G>T | Intron 5 |  |  | Predicted exonic splicing enhancers (PESE) |
|  |  |  |  | octamer motif from *Zhang & Chasin* and SP |
|  |  |  |  | protein SC35 binding site (ESE Finder matrices) |
|  |  |  |  | broken |
| g.45599001G>A | Intron 4 | Hepatocyte nuclear factor 3-alpha (HNF- | hsa-miR-3613-5p motif |  |
|  |  | 3alpha) binding site created | removed |  |
| g.45581730T>C | Intron 10 | Pituitary-specific factor 1(POU1F1) | hsa-miR-548n motif |  |
|  |  | binding site created | created |  |
| g.45586682G>A | Intron 8 | AR (androgen receptor) binding site | hsa-miR-4760-5p motif |  |
|  |  | created | created |  |
| g.45576724T>G | Intron 11 | GR (glucocorticoid receptor) binding site |  |  |
|  |  | created |  |  |
| g.45599377G>A | Intron 3 | POU2F1(POU class 2 transcription factor | hsa-miR-5685 motif | Putative exonic splicing silencer (PESS) octamer |
|  |  | 1) binding site created | created | motif from *Zhang & Chasin* created |
| g.45594075C>T | Intron 5 |  | hsa-miR-640 motif created | Potentially new acceptor splice sites created |
| g.45586821C>T | Intron 8 | ER-alpha/ER-beta (estradiol receptor- |  | Predicted exonic splicing enhancers (PESE) |
|  |  | alpha/beta )binding site removed |  | octamer motif from *Zhang & Chasin* broken |
| g.45581794A>G | Intron 10 | POU2F1(POU class 2 transcription factor |  |  |
|  |  | 1) binding site removed |  |  |
| g.45581388delTTAA | Intron 11 | Deletion: Cdx-3 (caudal related protein | Insertion: hsa-miR-2054 |  |
|  |  | 3) binding site created | motif removed |  |
| g.45586245T>C | Intron 8 | C/EBPbeta (CCAAT/enhancer-binding |  | Potentially new acceptor splice site created |
|  |  | protein beta) binding site removed |  |  |

**Table S5. Predicted influence of EBV-PAT-associated polymorphisms within *PLCz1* on transcription factor (TF) binding sites, microRNA (miRNA) and splice-site modifying motifs.**

**Table S5 continued.**

| SNP | Position in gene | TF binding site | miRNA motif | Splice-modifying motif |
| --- | --- | --- | --- | --- |
| g.45576460T>G | Intron 11 |  | hsa-miR-195-5p motif | Predicted exonic splicing silencer (PESS) |
|  |  |  | created | octamer motif from *Zhang & Chasin* and silencer |
|  |  |  |  | motif 3 from *Sironi et al.* created |
| g.45586601G>A | Intron 8 | MyoD (myogenic regulatory factors D) | hsa-miR-1266-5p motif | Predicted exonic splicing enhancers (PESE) |
|  |  | binding site removed | created | octamer motif from *Zhang & Chasin* broken |
| g.45612878T>C | 5’promotor region |  | hsa-miR-5693 motif | SP protein SF2/ASF binding site (ESE Finder |
|  |  |  | created | matrices) and predicted exonic splicing |
|  |  |  |  | enhancers (PESE) octamer motif from *Zhang &* |
|  |  |  |  | *Chasin* created |
