# Supplementary material for: Genome-Wide Association Study Identifies Phospholipase C zeta 1 (PLCz1) as a Stallion Fertility Locus in Hanoverian Warmblood Horses
Source: PLoS One. 2014 Oct 29;9(10):e109675. doi: 10.1371/journal.pone.0109675 (PMC4212906; doi:10.1371/journal.pone.0109675)
Supplement: Table S6 — Primer sequences and their positions, product sizes and annealing temperatures (AT) for sequencing the equine genomic DNA and cDNA of PLCz1 and CAPZA3 . (DOCX) [file pone.0109675.s011.docx]

**Table S6. Primer sequences and their positions, product sizes and annealing temperatures (AT) for sequencing the equine genomic DNA and cDNA of *PLCz1* and *CAPZA3*.**

| Gene | Gene region | Forward primer (5’-3’) | Reverse primer (5’-3’) | Product size (bp) | AT (°C) | Name | |
| --- | --- | --- | --- | --- | --- | --- | --- |
| *PLCz1* | Exon 1-6 | TAGCTTCCTGAACAGCCTTC | CCAGCAGTCAATTTCCAGAC | 917 | 57 | cPLCz_1 | |
| *PLCz1* | 5´UTR-Exon 4 | CAGACCAAAAGAAAAATATGACG* | CTTCTCTGTGTGCAATAATTCG* | 467 | 58 | cPLCz_1a | |
| *PLCz1* | Exon 1-3 | TTCTTCAAAAGTGGTGTGTCC* | TCTTTCCACCTCTAAAGTCATCC* | 365 | 58 | cPLCz_1b | |
| *PLC1z* | Exon 1-4 | TAGCTTCCTGAACAGCCTTC | CTCAGTAACTTCTTCTCTGTGTGC | 563 | 57 | cPLCz_1c | |
| *PLCz1* | Exon 2-6 | GCAAGGAGAAACAGAACAGC | CATATTTGCGTATCGCTTGG | 737 | 59 | cPLCz_1d | |
| *PLCz1* | Exon 5-9 | TTAAGCAAGCACACCAGATG | TTCTGAATTTCTCGGCTTTC | 804 | 57 | cPLCz_2 | |
| *PLCz1* | Exon 7-12 | TGCTCGATGAGTTTCTTGAC | ACACGAGTCTGATGTTTTGC | 855 | 56 | cPLCz_3 | |
| *PLCz1* | Exon 9-14 | TCTATTGGGGAGTCAAAAGC | TTGGCTGTTTTATTGAGAGG | 754 | 56 | cPLCz_4 | |
| *PLCz1* | Exon 1 | CCTGGAGGAGCCTGTATTAAC | GCAGCTAGGCAGCTATTCG | 558 | 58 | gPLCz Ex1 | |
| *PLCz1* | Exon 2 | TAGAGCACAGGGACCACATC | CTGGGCATGAGAGAGAAAAC | 690 | 57 | gPLCZ_Ex2 | |
| *PLCz1* | Exon 3 | CACCTACCTCCCAATTCCAG | ACTCGTGGTCCTTTAGGTTG | 729 | 58 | gPLCZ_Ex3 | |
| *PLCz1* | Exon 4 | TGTGCAATTGAAAGACAACC | AAAGAAAGGAGCTGGACAATG | 738 | 57 | gPLCZ_Ex4 | |
| *PLCz1* | Exon 5 | TTTTCCAAAGAAAAATTGGAAATAG | ATTCAGAGGGCCAATGACTG | 608 | 57 | gPLCZ_Ex5 |  |

**Table S6 continued.**

| Gene | Gene region | Forward primer (5’-3’) | Reverse primer (5’-3’) | Product size (bp) | AT^a^ (°C) | Name |  |
| --- | --- | --- | --- | --- | --- | --- | --- |
| *PLCz1* | Exon 6 | GACAGCAAGTGTGTCAATGG | GATTTCACTGCCTACTGATGG | 626 | 57 | gPLCz_Ex6 | |
| *PLCz1* | Exon 7 | GCTGGATGCTTCAGTTGTTT | ATTTTGGACTCCTGGGATCT | 649 | 57 | gPLCz_Ex7 | |
| *PLCz1* | Exon 8 | TTGCATAGTGCTTTGTGAAATG | AAAAACAGCGCAGGAAAAGA | 719 | 57 | gPLCz_Ex8 | |
| *PLCz1* | Intron 8 | CAATCAAGAAAAGGAAATGGAG | TGAACCTGAAAGCATATAAAACAG | 682 | 57 | gPLCz_Int8a | |
| *PLCz1* | Intron 8 | CTTGTTTTCAGGGAGATCAGAC | TTACTATTTCCTTTTCCCCAAAG | 715 | 57 | gPLCz _Int8b | |
| *PLCz1* | Intron 8 | TTTGTGCATTGGCTATAATCC | TGCTTTTGACTCCCCAATAG | 768 | 57 | gPLCz_ Int8c | |
| *PLCz1* | Exon 9 | TGACCGAGCAAATTCTTTTTG | CCAATTTTGACACCTTTGCAG | 824 | 59 | gPLCz_Ex9 | |
| *PLCz1* | Exon 10 | GTGAAACTAATTTGGATGTGAAAA | CCCATGCTAACTCCATGAAT | 651 | 57 | gPLCz_Ex10 | |
| *PLCz1* | Exon 11 | GCCCTGCCTGTTTCTTACC | AAAATACCACCCACGTATAGTACC | 766 | 59 | gPLCz_Ex11 | |
| *PLCz1* | Exon 12 | GCCTGACTTATAGTAGAAAGTGACTG | CACAGAGGCTAGAAGCCAAG | 843 | 57 | gPLCz_Ex12 | |
| *PLCz1* | Exon 13 | TTTGTGGCTCAGTTTTCTCA | AGTCTCCCCAGCAATCCTAC | 659 | 57 | gPLCz_Ex13 | |
| *PLCz1* | Exon 14 | CAGAGGGTGGGGACTAATTT | ACAAGCAAGTTGGAAGAGCA | 610 | 58 | gPLCz_Ex14 | |
| *CAPZA3* | Exon 1 | GGAGACTCAGAGCCTGCCAG | ATCCATTTAGATTGCCAAAGG | 680 | 58 | cCAPZA3_1a | |
| *CAPZA3* | Exon 1 | GGAGACTCAGAGCCTGCCAG | CAATTACCAGGAGCAAATCG | 1077 | 58 | cCAPZA3_1b | |

**Table S6 continued.**

| Gene | Gene region | Forward primer (5’-3’) | Reverse primer (5’-3’) | Product size (bp) | AT^a^ (°C) | Name |
| --- | --- | --- | --- | --- | --- | --- |
| *CAPZA3* | Exon 1 | TAACACCTTGATGGGAAAGG | CAATTACCAGGAGCAAATCG | 1010 | 58 | cCAPZA3_1c |
| *CAPZA3* | Exon 1 | TCATTGAAAGTTCCACACTTCAT | GTATCTCTAGTGGCTTCTCAACG | 1197 | 58 | gCAPZA3_Ex1 |
| *CAPZA3* | Exon 1 | TCATTGAAAGTTCCACACTTCAT | GAAAATAACCACTCCCCAGAA | 1170 | 58 | gCAPZA3_Ex1a |
| *CAPZA3* | Exon 1 | TCATTGAAAGTTCCACACTTCAT | ATCCATTTAGATTGCCAAAGG | 730 | 58 | gCAPZA3_Ex1b |
| *CAPZA3* | Exon 1 | TGCTTCAAAACCAGCTAAAAG | GTATCTCTAGTGGCTTCTCAACG | 702 | 58 | gCAPZA3_Ex1c |
