# Supplementary material for: Genome-Wide Association Study Identifies Phospholipase C zeta 1 (PLCz1) as a Stallion Fertility Locus in Hanoverian Warmblood Horses
Source: PLoS One. 2014 Oct 29;9(10):e109675. doi: 10.1371/journal.pone.0109675 (PMC4212906; doi:10.1371/journal.pone.0109675)
Supplement: Table S7 — Genotyping techniques used for validation of polymorphisms in 237 Hanoverian stallions. All single nucleotide polymorphisms (SNPs) genotyped using RFLPs (restriction fragment length polymorphisms) or IRD (infrared dye) labeled gel electrophoresis are given. (DOCX) [file pone.0109675.s012.docx]

**Table S7. Genotyping techniques used for validation of polymorphisms in 237 Hanoverian stallions.** All single nucleotide polymorphisms (SNPs) genotyped using RFLPs (restriction fragment length polymorphisms) or IRD (infrared dye) labeled gel electrophoresis are given.

| Gene | Polymorphism ID | Primer (5‘-3‘) | Method of | Product | TA | Restriction enzyme, |
| --- | --- | --- | --- | --- | --- | --- |
|  |  |  | genotyping | size (bp) | (°C) | TaqMan probe or IRD |
|  |  |  |  |  |  | labeling |
| *CAPZA3* | g.45613675T>C | F: GGAGACTCAGAGCCTGCCAG | RFLP | 1077 | 58 | *BmrI* |
|  |  | R: CAATTACCAGGAGCAAATCG |  |  |  |  |
| *PLCz1* | g.45612878T>C | F: TAAGTGACATGTTTGGTTGCAGCAGACTCA | Missmatch PCR- RFLP | 242 | 58 | *DdeI* |
|  |  | R: GTGGAAGGCTTATTTATCTCTCGGCTATCC |  |  |  |  |
| *PLCz1* | g.45612721C>G | F: CTCTGAGTCTCCTGATGAAGTCATAAGGAA | RFLP | 337 | 62 | *NlaIV* |
|  |  | R: GCAGCTAGGCAGCTATTCG |  |  |  |  |
| *PLCz1* | g.45610678delA | F: CACCTACCTCCCAATTCCAG | RFLP | 729 | 58 | *Cac8I* |
|  |  | R: ACTCGTGGTCCTTTAGGTTG |  |  |  |  |
| *PLCz1* | g.45599377G>A | F: TTTTCATATTCACAATGTAAGCCCACACGT | Missmatch PCR- RFLP | 246 | 59 | *BmgBI* |
|  |  | R: TCAGAGGCAATAGTTTTAGTCATCTC |  |  |  |  |
| *PLCz1* | g.45599207G>A | F: CATATTCTGAAAATCGGAAGATTCTTTTGGAA | Custom TaqMan® | 108 | 60 | CTGCTCTTGTATCA AAA_ |
|  |  | R: CAATGATCTCAGAGGCAATAGTTTTAGTCA | SNP Genotyping-Assay |  |  | FAM_TM_/CTGCTCTTGTGTC |
|  |  |  |  |  |  | AAAA_ VIC^®^ |
| *PLCz1* | g.45595295A>T | F: TTTTCCAAAGAAAAATTGGAAATAG | RFLP | 608 | 57 | *NdeI* |
|  |  | R: ATTCAGAGGGCCAATGACTG |  |  |  |  |
| *PLCz1* | g.45595152C>T | F: TTTTCCAAAGAAAAATTGGAAATAG | RFLP | 608 | 57 | *EarI* |
|  |  | R: ATTCAGAGGGCCAATGACTG |  |  |  |  |
| *PLCz1* | g.45594143G>A | F: CAACTTGTCTTTATTATGGATTTATTGAGAC | Missmatch PCR-RFLP | 230 | 58 | *BsmAI* |
|  |  | R: GCTTGCTGGTGAGTGTATAACC |  |  |  |  |

**Table S7 continued.**

| Gene | Polymorphism ID | Primer (5‘-3‘) | Method of | Product | TA | Restriction enzyme, |
| --- | --- | --- | --- | --- | --- | --- |
|  |  |  | genotyping | size (bp) | (°C) | TaqMan probe or IRD |
|  |  |  |  |  |  | labeling |
| *PLCz1* | g.45586159G>A | F: TGACCGAGCAAATTCTTTTTG | RFLP | 824 | 59 | *AluI* |
|  |  | R: CCAATTTTGACACCTTTGCAG |  |  |  |  |
| *PLCz1* | g.45586134G>C | F: TGACCGAGCAAATTCTTTTTG | RFLP | 824 | 59 | *HpyCH4III* |
|  |  | R: CCAATTTTGACACCTTTGCAG |  |  |  |  |
| *PLCz1* | g.45581388delTTAA | F: CCCAAATAAAGCACCAATAGAC | Product size determination | 220 | 59 | IRD; DY-682-labeling |
|  |  | R: AAAATACCACCCACGTATAGTACC | (LI-COR 4300) |  |  |  |
| *PLCz1* | g.45581794A>G | F: ACCTTCATACATTAATATAGAAATGGTCAA | Missmatch PCR-RFLP | 280 | 55 | *HincII* |
|  |  | R. TTTCAAAACATATCCAGAACCAC |  |  |  |  |
| *PLCz1* | g.45581730T>C | F: GCCCTGCCTGTTTCTTACC | RFLP | 762 | 59 | *HpyCH4V* |
|  |  | R: AAAATACCACCCACGTATAGTACC |  |  |  |  |
| *PLCz1* | g.45599001G>A | F: TCTGGCTAATAGCAAATATTGGCAGTTGTA | Missmatch PCR-RFLP | 336 | 58 | *CviQI* |
|  |  | R: CCTAGAATTGTTTTCGTTCAGC |  |  |  |  |
